# Supplementary material for: Differences in aortic valve area measured on cardiac CT and echocardiography in patients with aortic stenosis
Source: PLoS One. 2023 Jan 20;18(1):e0280530. doi: 10.1371/journal.pone.0280530 (PMC9858019; doi:10.1371/journal.pone.0280530)
Supplement: S1 File — (DOCX) [file pone.0280530.s001.docx]

**Supplementary table 1.** Baseline echocardiographic findings based on AS severity and its subgroups.

| Echocardiography | High gradient  severe AS (n = 438) | | | Classic LF-LG AS  (n = 18) | | | Paradoxical LF-LG AS  (n = 55) | | | Moderate AS  (n = 24) | | | |
| --- | --- | --- | --- | --- | --- | --- | --- | --- | --- | --- | --- | --- | --- |
|  | Concordant | Discordant |  | Concordant | Discordant |  | Concordant | Discordant |  | Concordant | Discordant |  |  |
|  | AVA_CT_  < 1.2 cm^2^ | AVA_CT_  ≥ 1.2 cm^2^ | *P* value | AVA_CT_  < 1.2 cm^2^ | AVA_CT_  ≥ 1.2 cm^2^ | *P* value | AVA_CT_  < 1.2 cm^2^ | AVA_CT_  ≥ 1.2 cm^2^ | *P* value | AVA_CT_  ≥ 1.2 cm^2^ | AVA_CT_  < 1.2 cm^2^ | *P* value |  |
| No. of patients* | 395 (90.2) | 43 (9.8) |  | 13 (72.2) | 5 (27.8) |  | 47 (85.5) | 8 (14.5) |  | 20 (83.3) | 4 (16.7) |  |  |
| LVEF, %^*^ | 60.3±10.1 | 60.8±8.9 | 0.72 | 36.9±11.2 | 33.8±8.0 | 0.59 | 62.8±5.4 | 61.5±4.6 | 0.53 | 53.2±12.4 | 66.3±4.6 | 0.05 |  |
| Peak velocity, m/s^*^ | 5.2±0.7 | 4.9±0.6 | 0.006 | 3.6±0.4 | 3.4±0.7 | 0.37 | 3.5±0.5 | 3.6±0.6 | 0.67 | 3.7±1.0 | 4.4±0.2 | 0.18 |  |
| Peak PG, mmHg^*^ | 109.8±31.0 | 96.9±22.4 | 0.001 | 53.5±11.7 | 48.0±17.0 | 0.44 | 49.5±16.0 | 53.4±16.9 | 0.53 | 61.1±27.1 | 75.3±15.2 | 0.33 |  |
| Mean PG, mmHg^*^ | 67.3±19.7 | 58.5±14.7 | 0.001 | 30.8±6.8 | 27.2±10.6 | 0.41 | 27.7±9.4 | 29.8±10.2 | 0.59 | 37.1±16.8 | 45.5±9.8 | 0.34 |  |
| LVMI, g/m^2*^ | 135.4±35.8 | 134.8±36.5 | 0.91 | 151.5±33.5 | 144.7±26.3 | 0.69 | 121.5±32.7 | 144.6±50.6 | 0.10 | 132.9±27.3 | 102.7±21.7 | 0.05 |  |
| AV VTI, cm^*^ | 127.2±25.7 | 101.0±16.5 | < 0.001 | 101.3±34.2 | 78.2±26.9 | 0.20 | 114.2±28.2 | 98.9±21.3 | 0.15 | 78.3±16.9 | 98.5±7.7 | 0.03 |  |
| LVOT VTI, cm^*^ | 21.3±4.1 | 22.6±3.6 | 0.04 | 16.0±4.8 | 16.2±3.8 | 0.92 | 21.4±3.6 | 19.7±3.7 | 0.24 | 22.8±4.8 | 24.8±5.1 | 0.45 |  |
| LVOT diameter, mm^*^ | 21.0±1.5 | 21.4±1.5 | 0.10 | 22.1±2.2 | 21.2±0.2 | 0.22 | 21.0±1.4 | 22.2±2.2 | 0.18 | 22.4±2.2 | 19.4±5.6 | .07 |  |
| LVOT diameter/BSA, mm^*^ | 12.9±1.3 | 12.3±1.0 | < 0.001 | 12.9±1.6 | 13.6±1.4 | 0.40 | 13.0±1.4 | 13.2±1.2 | 0.75 | 12.8±1.3 | 11.1±2.8 | 0.06 |  |
| AVA_echo_, mm^2*^ | 59.0±13.6 | 80.4±8.8 | < 0.001 | 61.1±12.8 | 81.9±9.1 | 0.005 | 68.2±13.6 | 77.3±12.7 | 0.08 | 115.4±16.0 | 107.2±0.36 | 0.33 |  |
| ESVI, mL/m^2*^ | 27.7±16.9 | 28.0±16.4 | < 0.92 | 63.1±30.0 | 64.4±20.0 | 0.92 | 23.4±10.6 | 30.7±14.5 | 0.10 | 37.2±21.6 | 17.5±5.2 | 0.09 |  |
| EDVI, mL/m^2*^ | 66.6±23.4 | 68.7±26.7 | 0.57 | 96.4±32.6 | 95.9±21.3 | 0.98 | 62.0±22.6 | 78.4±31.5 | 0.08 | 74.9±27.6 | 51.4±10.1 | 0.11 |  |
| SAC, mL/m^2^/mmHg^*^ | 0.8±0.3 | 0.8±0.3 | 0.11 | 0.7±0.3 | 0.6±0.1 | 0.52 | 0.7±0.3 | 1.0±0.5 | 0.21 | 0.7±0.23 | 0.7±0.2 | 0.63 |  |
| Valvulo-arterial impedance (Zva), mmHg/mL/m^2*^ | 5.4±1.6 | 5.2±1.7 | 0.51 | 4.7±1.7 | 58. ±2.2 | 0.82 | 4.5±1.4 | 3.8±1.5 | 0.21 | 4.9±1.6 | 5.3±1.1 | 0.69 |  |

Note.–Data are mean and standard deviation. ^*^Data are numbers and percentages in parentheses. AS, aortic stenosis; AVA, aortic valve area; BSA, body surface area; EDVI, end-diastolic volume index; ESVI, end-systolic volume index; LF-LG, low-flow and low-gradient; LVEF, left ventricular ejection fraction; LVMI, left ventricular mass index; LVOT, left ventricular outflow tract; PG, pressure gradient; SAC, systemic arterial compliance; VTI, velocity time integral.

**Supplementary table 2.** Clinical and CT characteristics of the concordant and discordant groups between AVA values measured by echocardiography and CT in moderate AS

| Characteristics | Concordant group  (n = 20) | Discordant group  (n = 4) | *P* value |
| --- | --- | --- | --- |
|  | AVA_echo_ ≥1.0 cm^2^ and  AVA_CT_ ≥1.2 cm^2^ | AVA_echo_ ≥1.0 cm^2^ and  AVA_CT_ <1.2 cm^2^ |  |
| Age, years^*^ | 67.00 (59.00–74.00) | 64.50 (53.00–74.50) | 0.67 |
| Male | 14 (70.00) | 3 (75.00) | > 0.99 |
| BSA, m^2*^ | 1. 75 (1.60–1.86) | 1.73 (1.65–1.84) | 0.94 |
| Hypertension | 16 (88.00) | 2 (50.00) | 0.25 |
| Atrial fibrillation | 4 (20.00) | 2 (50.00) | 0.25 |
| PCI or CABG | 5 (25.00) | 1 (25.00) | 0.62 |
| Rheumatic valvular disease | 4 (20.00) | 1 (25.00) | > 0.99 |
| Echocardiography |  |  |  |
| LVEF, %^*^ | 57.03(41.27–64.13) | 67.91 (63.14–69.38) | 0.02 |
| Peak velocity, m/s^*^ | 3.85 (2.90–4.35) | 4.35 (4.05–4.70) | 0.17 |
| Peak PG, mmHg^*^ | 65.00 (38.25–81.50) | 75.00 (63.50–87.00) | 0.29 |
| Mean PG, mmHg^*^ | 37.00 (22.50–48.50) | 45.50 (38.0–53.00) | 0.28 |
| LVMI, g/m^2*^ | 135.05 (111.20–158.35) | 102.3 (84.25–121.20) | 0.05 |
| AV VTI, cm^*^ | 74.91 (65.65–88.60) | 94.90 (94.25–102.65) | 0.02 |
| LVOT VTI, cm^*^ | 23.05 (20.38–26.50) | 25.05 (20.45–29.20) | 0.54 |
| LVOT diameter, mm^*^ | 22.00 (21.00–23.75) | 20.50 (15.85–22.95) | 0.31 |
| LVOT diameter/BSA, mm^*^ | 13.19 (11.72–13.71) | 11.48 (9.16–12.99) | 0.22 |
| AVA_echo_, mm^2*^ | 109.16 (104.47–122.75) | 107.26 (106.91–107.51) | 0.59 |
| ESVI, mL/m^2*^ | 31.91 (19.71–52.98) | 16.35 (14.41–20.61) | 0.03 |
| EDVI, mL/m^2*^ | 67.58 (56.86–95.07) | 53.40 (45.31–57.59) | 0.05 |
| SAC, mL/m^2^/mmHg^*^ | 0.74 (0.54–0.86) | 0.66 (0.53–0.78) | 0.64 |
| Valvulo-arterial impedance (Zva), mmHg/mL/m^2*^ | 4.39 (3.72–5.86) | 5.18 (4.53–6.00) | 0.33 |
| CT findings |  |  |  |
| Valve morphology |  |  | 0.22 |
| Tricuspid (%) | 12 (60.00) | 2 (50.00) |  |
| Bicuspid with raphe (%) | 3 (15.00) | 2 (50.00) |  |
| Bicuspid without raphe (%) | 5 (25.00) | 0 (0) |  |
| LVOT mean diameter^*^ | 25.73 (24.03–27.83) | 23.35 (22.49–24.41) | 0.09 |
| AVA calcium score^†^ | 1610.30 (714.20–2544.00) | 787.45 (394.95–1537.85) | 0.25 |
| AVA_plani_, mm^2*^ | 117.52 (99.33–134.00) | 144.45 (130.97–157.80) | 0.10 |
| AVA_CT_, mm^2*^ | 151.45 (140.61–179.24) | 108.88 (103.07–114.68) | 0.002 |
| Aortic annulus |  |  |  |
| Circularity, %^*^ | 0.81 (0. 77–0.85) | 0.83 (0.76–0.85) | 0.88 |
| Maximal dimeter, mm^*^ | 29.70 (26.66–31.25) | 25.95 (24.65–27.85) | 0.08 |
| Mean diameter, mm^*^ | 26. 51 (24.85–28.03) | 23.10 (21.71–25.67) | 0.06 |
| Perimeter, mm^*^ | 85.20 (76.69–89.55) | 73.05 (68.80–79.20) | 0.02 |
| Area, mm^2*^ | 534.50 (447.78–612.90) | 402.22 (349.20–474.62) | 0.04 |
| Sinus of Valsalva diameter, mm^*^ | 36.45 (33.32–38.78) | 38.02 (34.35–40.23) | 0.76 |
| Sinotubular junction diameter, mm^*^ | 30.20 (28.53–34.05) | 33.74 (30.25–37.64) | 0.25 |
| Ascending aorta tubular portion, mm^*^ | 38.70 (36.10–44.85) | 37.20 (34.03–47.25) | 0.51 |
| Surgical valve size, mm^*^ | 23.00 (21.00–25.00) | 23.00 (21.00–24.50) | 0.94 |
| Postoperative findings |  |  |  |
| LVEF, %^*^ | 61.30 (46.53–64.60) | 66.75 (62.72–68.59) | 0.14 |
| Peak velocity, m/s^*^ | 3.0 (2.00–3.00) | 3.00 (2.50–3.00) | 0.82 |
| Peak PG, mmHg^*^ | 26.00 (22.50–37.50) | 25.00 (17.50–30.00) | 0.56 |
| Mean PG, mmHg^*^ | 14.50 (12.00–20.50) | 13.50 (9.50–15.50) | 0.44 |
| LVMI, g/m^2*^ | 117.85 (95.85–139.35) | 76.90 (71.20–89.65) | 0.02 |
| MACCE | 4 (20.00) | 0 (0) | > 0.99 |
| Overall mortality | 3 (15.00) | 0 (0) | > 0.99 |

Note.–Data are numbers and percentages in parentheses. ^*^Data are median and interquartile range in parentheses. AS, aortic stenosis; AVA, aortic valve area; BSA, body surface area; CABG, coronary artery bypass graft; EDVI, end-diastolic volume index; ESVI, end-systolic volume index; LFLG, low-flow and low-gradient; LVEF, left ventricular ejection fraction; LVMI, left ventricular mass index; LVOT, left ventricular outflow tract; MACCE, major adverse cardiac and cerebrovascular event; PCI, percutaneous coronary intervention; PG, pressure gradient; SAC, systemic arterial compliance; VTI, velocity time integral.
