# Supplementary material for: Differences in aortic valve area measured on cardiac CT and echocardiography in patients with aortic stenosis
Source: PLoS One. 2023 Jan 20;18(1):e0280530. doi: 10.1371/journal.pone.0280530 (PMC9858019; doi:10.1371/journal.pone.0280530)
Supplement: Attachment — Submitted filename: Peer Review - PLOS ONE -Ossama Abou Hassan Oct 2022.docx [file pone.0280530.s002.docx]

The manuscript entitled "Differences in Aortic Valve Area Measured on Cardiac CT and Echocardiography in Patients with Aortic Stenosis" by Koo. et al. aimed to compare the aortic valve area AVA measured on CT and echocardiography, and demonstrate the factors that lead to discordant AVA readings between CT and echocardiography. From the 781 retrospectively selected patients, 95.51% were documented to have echocardiographic severe aortic stenosis and 10.96% of those had discordance between AVA_echo_ and AVA_CT_. Larger BSA, AVA_echo_, tricuspid AV morphology and indexed annulus area were associated with AVA discordance between echo and CT.

The authors have done a lot of work in collating this information and improving our knowledge in multimodality AS classification with few clarifications requested.

1. It should be highlighted in the text that the main comparison between AVA_echo_ and AVA_CT_ utilizes continuity equation for both and thus are comparisons of an echo solely or a hybrid approach. This said, a hybrid approach measurement can also be simplified to the comparison of annular area in both modalities.
2. Furthermore, the discussion section sheds less light on AVA_plani_ as a comparison and thus is confusing as the norm of a CT approach is usually to check planimetry area measurements. If that is not the case, it is then important to emphasize the differences within CT measurements whether measurement have been taken by the hybrid approach or by planimetry.

Comments to the Editors:
This paper is simple but important in our growing understanding of disparities in intermodality aortic stenosis severity classification. The authors’ findings can help in the daily clinical evaluation of patients with severe aortic stenosis and as such it is my judgement that this may be accepted after the minor revisions suggested above.
